# Viral RNA Load in Symptomatic and Asymptomatic COVID-19 Omicron Variant-Positive Patients

**DOI:** 10.1155/2022/5460400

**Published:** 2022-08-29

**Authors:** Qian Wu, Lixia Shi, Haomin Li, Shuping Huang, Hongwei Li, Li Li, Jin Han, Qi Wu, Zhengcun Pei

**Affiliations:** ^1^Haihe Clinical School, Tianjin Medical University, Tianjin, China; ^2^Department of Respiratory Medicine, Haihe Hospital, Tianjin University, Tianjin, China; ^3^Tianjin Institute of Respiratory Diseases, Tianjin, China; ^4^Department of Medicine, Haihe Hospital, Tianjin University, Tianjin, China; ^5^Department of Orthopedics, Tianjin Hospital, Tianjin, China; ^6^Department of Respiratory Medicine, Tianjin Medical University General Hospital, Tianjin, China; ^7^Academy of Medical Engineering and Translational Medicine, Tianjin University, Tianjin, China

## Abstract

**Objectives:**

Viral load is important when evaluating viral transmission potential, involving the use of a polymerase chain reaction (PCR) cycle threshold (Ct) value. We aimed to analyze the PCR Ct values of respiratory tract samples taken from patients with severe acute respiratory syndrome coronavirus 2 (SARS-CoV-2) Omicron variant strains to evaluate these strains' viral dynamics.

**Methods:**

This study comprised 361 patients. The Ct values of SARS-CoV-2-related respiratory samples were compared between symptomatic and asymptomatic patients.

**Results:**

The median (25^th^ percentile and 75^th^ percentile) nasopharynx and oropharynx SARS-CoV-2 Ct values were 30.5 (24.5–35.0) and 34.5 (30.0–37.0) in the symptomatic group, respectively, and 27.8 (23.4–34.5) and 33.5 (26.0–35.0) in the asymptomatic group, respectively, without significance. In the symptomatic group, subgroup analyses according to age showed the mean nasal Ct value for patients aged >18 years was 29.0 (23.5–34.5), which was significantly lower than that of patients aged 0–4 years and 5–13 years (36.0 (30.5–38.0) and 34.5 (31.0–39.0), respectively). The nasal Ct value for asymptomatic patients aged >18 years was 25.5 (20.9–28.4), which was significantly lower than of patients aged 5–13 years (34.5 (25.6–36.4)).

**Conclusion:**

Our findings suggest that the viral loads of asymptomatic and symptomatic patients did not differ significantly. However, adults infected with SARS-CoV-2 had higher nasal viral loads that those of young children.

## 1. Introduction

The Omicron variant of severe acute respiratory syndrome coronavirus 2 (SARS-CoV-2) (B.1.1.529) was first reported in November 2021. On 26 November 2021, the World Health Organization (WHO) designated B.1.1.529 as a variant of concern (VOC) [1]. The Omicron VOC genome contains >50 mutations, and the spike protein has 37 mutations, many of which are associated with increased infectivity and avoidance of immune responses to previous VOCs such as the Alpha and Delta variants [2]. The international spread of the Omicron variant has caused a surge in infection rates in some regions, with major repercussions. The first case of Omicron in China was reported on January 8, 2022, in Tianjin. Currently, there is very limited information on transmissibility and vaccine efficacy in relation to the Omicron variant. We reviewed the clinical records of symptomatic and asymptomatic patients, assessed their cycle threshold (Ct) values for SARS-CoV-2 nucleic acid, and analyzed differences in viral loads in the upper respiratory tracts.

## 2. Materials and Methods

### 2.1. Study Participants

We conducted a retrospective cross-sectional study of 361 patients at the Tianjin Haihe Hospital, China, from January 8, 2022, to January 25, 2022. The patients had tested positive for SARS-CoV-2 and were classified as Omicron variant-positive using whole genome sequencing. Patients with one or more symptoms of coronavirus disease 2019 (COVID-19), namely, fever, cough, fatigue, decreased or loss of ability to smell and taste, nasal congestion, runny nose, sore throat, conjunctivitis, myalgia, and diarrhea, together with a positive SARS-CoV-2 nucleic acid test, were defined as symptomatic. Patients with a positive SARS-CoV-2 nucleic acid test, but with no COVID-19-related symptoms at the time of testing, were defined as asymptomatic.

### 2.2. Data Collection

We collected clinical information concerning each patient, including age, sex, comorbidities, symptoms, nucleic acid test Ct value, nucleic acid-negative conversion time, and vaccination status.

The COVID-19 kit we employed uses two targets: the SARS-CoV-2 RdRp (ORF1ab) gene and the SARS-CoV-2 N gene. Fluorescence quantitative PCR was used to detect the number of cycles (Ct value) for fluorescence to reach a preset threshold. Mean Ct values of symptomatic and asymptomatic patients in different age groups were compared. Subgroups in the symptomatic and asymptomatic groups were categorized according to age as follows: 0–4 years, 5–13 years, 14–18 years, and >18 years. If the test had multiple target results, an average of the Ct values was sampled. If only one target was positive, a single Ct value was used. All symptomatic and asymptomatic patients were tested for respiratory tract samples (nasopharyngeal or oropharyngeal).

As this study involved a retrospective analysis, the Ethics Review Board at our institution waived the requirement for informed patient consent.

### 2.3. Statistical Analysis

IBM SPSS Statistics for Windows, version 19.0 (IBM Corp., Armonk, NY, USA) software, was used for the statistical analyses. Continuous variables are presented with the median (25th percentile, 75th percentile) due to skewed distributions and categorical variables are presented as *n* (%). A comparison of rates was performed using a Chi-square test, and a Mann–Whitney *U* test or a Kruskal–Wallis test was used for comparison between the groups, as appropriate. A *P* value <0.05 indicated a statistical significance.

## 3. Results

This study included 22 and 339 asymptomatic and symptomatic patients, respectively. Of 339 symptomatic patients, 188 had mild symptoms (55.2%) and 151 were moderate cases (44.8%). Among 22 patients in the symptomatic group, 16 (72.6%) were aged >18 years. In the asymptomatic group, 11 (50%) patients were aged >18 years and 11 (50%) patients were aged 5–13 years. There were 20 (90.9%) and 299 (88.2%) vaccinated patients in the asymptomatic and symptomatic groups, respectively. In the symptomatic group, the most prevalent comorbidity was hypertension (*n* = 57 patients; 16.8%), followed by diabetes mellitus (*n* = 21 patients; 6.2%) and coronary heart disease (*n* = 14 patients; 4.1%). In the asymptomatic group, the most prevalent comorbidity was hypertension (*n* = 4 patients; 18.2%), followed by coronary artery disease (*n* = 2 patients; 9.1%) and diabetes mellitus (*n* = 1 patient; 4.5%). No statistically significant differences in terms of sex, vaccination status, and the presence of comorbidities were observed between the two groups (Table 1).

The mean Ct value of SARS-CoV-2 for the nasopharynx samples in the symptomatic group was 30.5 (24.5–35), whereas it was 27.8 (23.4–34.5) in the asymptomatic group. There was no statistically significant difference between the two groups. The mean Ct values of SARS-CoV-2 for the oropharynx samples of the symptomatic and asymptomatic groups were 34.5 (30–37) and 33.5 (26–35), respectively. No statistically significant difference was found between the two groups (Table 2).

**Figure 1:**
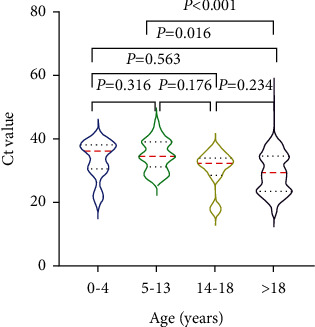
Ct values of nasopharyngeal nucleic acid tests in symptomatic patients stratified by age.

**Figure 2:**
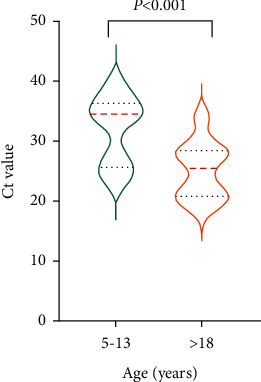
Ct values of nasopharyngeal nucleic acid tests in asymptomatic patients stratified by age.

Analyses of subgroups classified according to age for the symptomatic group showed that the SARS-CoV-2 Ct value of nasopharyngeal samples from patients aged >18 years was 29 (23.5–34.5), which was significantly lower than those of the 0–4 (36 (30.5–38)) and 5–13-year-old groups (34.5 (31–39)) (Figure 1). The mean Ct value for patients aged >18 years in the asymptomatic group was 25.5 (20.9–28.4), which was significantly lower than that of patients aged 5–13 years (34.5 (25.6–36.4)) (Figure 2).

**Figure 3:**
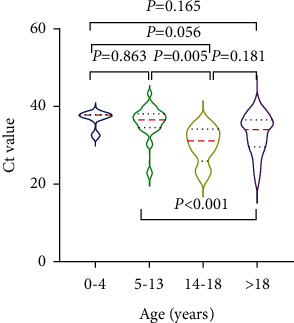
Ct values of oropharyngeal nucleic acid tests in symptomatic patients stratified by age.

**Figure 4:**
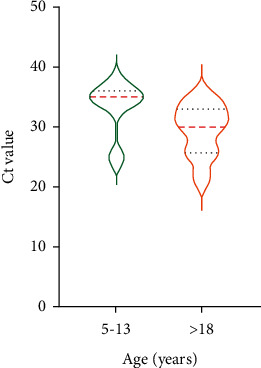
Ct values of oropharyngeal nucleic acid tests in asymptomatic patients stratified by age.

For the symptomatic group, SARS-CoV-2 Ct values for oropharyngeal samples were the lowest in the subgroup of 14–18 year-old individuals (31 (25.8–34)), compared with the subgroup of patients aged >18 years (34 (29.5–36.5)) and the subgroup of 5–13 year-old individuals (36.5 (34.5–38)) (Figure 3). The mean Ct value for oropharyngeal samples for the asymptomatic group was 30 (25.8–33) for the subgroup aged >18 years, which did not differ from the mean Ct value for the subgroup of patients aged 5–13 years old (35 (31.4–36)) (Figure 4).

The Ct values obtained using nasopharyngeal and oropharyngeal swabs from both the symptomatic and asymptomatic patients showed no statistically significant difference between the vaccinated and unvaccinated participants (Table 3).

## 4. Discussion

The WHO has warned that the Omicron variant of SARS-CoV-2 has a very high risk of infection. Following D614 G, Beta/Gamma, and Delta VOCs, SARS-CoV-2 Omicron variants may act as catalysts for a fourth wave of COVID-19 outbreak. To explore the viral dynamics of Omicron variants in different age groups, this study analyzed the Ct values of SARS-CoV-2 nucleic acid tests in respiratory samples obtained from symptomatic and asymptomatic individuals infected with the Omicron variant in China. Differences in the viral load between nasopharyngeal and oropharyngeal sites were analyzed.

Most of the patients in the symptomatic group and 50% of the patients in the asymptomatic group were over 18 years old. Notably, most individuals infected with the Omicron variant in our institution were symptomatic adult patients. Our data showed that the most prevalent comorbidities among Omicron variant-positive patients were hypertension, diabetes mellitus, and coronary artery disease, which are similar to the comorbidities found to be the most prevalent among patients infected with previous ancestral strains of SARS-CoV-2 [3]. In this study, the nucleic acid sampling time from onset of the infection was 5 days for the symptomatic patients and 4 days for the asymptomatic patients. A study conducted in Japan found that the viral RNA load was the highest on 3–6 days after diagnosis or symptom onset [4]. The National Institute of Infectious Diseases used quantitative reverse transcription PCR and virus isolation tests to quantify SARS-CoV-2 RNA in 83 respiratory specimens from 21 patients. The amount of viral RNA was found to peak 3–6 days after diagnosis or symptom onset and then decline gradually [5]. Based on studies concerning viral shedding time, the nucleic acid sampling time for the present study was chosen at the peak of viral RNA volume. Ct values, which are used to assess viral load in patients with SARS-CoV-2, have been reported to vary widely across populations and time periods [6]. Kociolek et al. found lower Ct values in the respiratory tract specimens of symptomatic children compared to those of asymptomatic children [7]. Data from Hurst et al. showed no significant differences in the viral load between symptomatic and asymptomatic children [7–9]. Studies investigating differences in viral load in adults showed a high viral load in asymptomatic adults [10, 11]. Our data suggest that there was no difference in the SARS-CoV-2 nucleic acid Ct values of nasopharyngeal and oropharyngeal swabs between the symptomatic and asymptomatic groups, suggesting that the capacity for virus detection was similar for these two sites. Because the viral load correlates with Ct values, similar Ct values for nasopharyngeal and oropharyngeal samples may also indicate that viral loads are similar at both these sampling sites.

For patients in different age subgroups, our study found that the nasopharyngeal Ct values of patients aged >18 years were significantly lower than those in other age subgroups, both in the symptomatic and in the asymptomatic groups. This finding suggests that the nasopharyngeal viral load may be higher in people aged >18 years. Demographic information showed that 72.6% of the symptomatic patients and 50% of the asymptomatic patients were aged >18 years. Patients aged 14–18 years accounted for 2.4% of the symptomatic group. Based on the above results, and from the perspective of the overall population, patients over the age of 14, especially those over 18 years of age, would appear to constitute the majority of patients diagnosed with the Omicron variant. Furthermore, these patients also have the highest viral load during the 4^th^ and 5^th^ days of illness. Compared with SARS-CoV, which replicates mainly in the alveolar epithelium, SARS-CoV-2 replicates extensively in both bronchial and alveolar epithelia, which, together with other factors, might explain its more efficient transmission [12]. For all the abovementioned reasons, patients aged >18 years should be carefully assessed on the 4^th^ and 5^th^ days of illness.

Although adult patients admitted to our institution had low Ct values and high viral loads, there were no severe cases in the adult population and the disease classification was mild or moderate. It has been reported that patients with preexisting cellular and innate immunity, nonneutralizing antibodies, and residual neutralizing antibodies may be protected from a severe disease [13]. In this study, the vaccination rate in both asymptomatic and symptomatic groups of patients was approximately 90% and the mean IgG levels were 31.64 and 20.37, respectively. We believe that the reason for the lack of severe infection in these patients may be related to vaccination and antibody protection. A study from South Africa also found that Omicron-positive patients had mild symptoms and did not require oxygen support [14], which accords with observations in our institution that patients with the Omicron variant did not have a severe disease.

The results of this study showed that the nucleic acid conversion time of the symptomatic and asymptomatic groups was 11.5 days and 12 days, respectively, with no statistically significant difference. Previous analyses of the negative nucleic acid transition time of 12,400 novel wild coronavirus strains found that the median interval between SARS-CoV-2 positive and negative tests was 14 days (8–19.25) [15]. In terms of the number of days for the nucleic acid test to turn negative, the time for Omicron-infected patients may be shorter compared to that of other strains. However, the sample size in this study was limited, warranting further investigation in studies with a larger sample size.

This study had some limitations. The collected samples lacked granular data on the days of infection and did not describe the curve of change in Ct values. Furthermore, although Ct values are representative of viral load, they may not correlate with the number of live viruses.

## 5. Conclusions

Our study suggested that both symptomatic and asymptomatic patients had the same viral load, with a similar infectivity potential. Adults infected with SARS-CoV-2 had a higher nasal viral load compared to that of young children. However, other studies have shown that symptomatic children have RNA levels comparable to or higher than adults [16–18]; therefore, screening of the child population should not be overlooked.

## Figures and Tables

**Table 1:** Comparison of asymptomatic and symptomatic groups by gender, age group, vaccination status, disease severity, antibody levels, time to negative nucleic acid test, and presence of comorbidities.

Variable	Asymptomatic (*n* = 22)	Symptomatic (*n* = 339)	*P*
*Sex*	—	—	—
Male	6 (27.3)	159 (46.9)	0.073
Female	16 (72.7)	180 (53.1)	

*Age (years)*	—	—	—
0–4	0	11 (3.2)	—
5–13	11 (50)	85 (23.5)	—
14–18	0	8 (2.4)	—
＞18	11 (50)	246 (72.6)	—

*Vaccination*			
Yes	20 (90.9)	299 (88.2)	0.967
No	2 (9.1)	40（11.8）	

*Severity*	—	—	—
Mild	—	188 (55.2)	—
Moderate	—	151 (44.8)	—

*SARS-CoV-2 antibodies*	—	—	—
IgM mean (range),S/CO	0.30 (0.09–0.92)	0.22 (0.09–0.52)	0.384
IgG mean (range),S/CO	31.64 (12.7–113.72)	20.37 (2.25–53.54)	0.068
Nucleic acid detection from the onset of time (days), mean (range)	5 (3.5, 6.5)	4 (3, 6)	0.317
Time to negative nucleic acid test (days), mean (range)	11.5 (10, 13)	12 (9.5, 13)	0.974

*Comorbidities*	—	—	—
Hypertension	4 (18.2)	57 (16.8)	1.0
Diabetes	1 (4.5)	21 (6.2)	1.0
Coronary artery disease	2 (9.1)	14 (4.1)	0.254
Cerebrovascular disease	0	11 (3.2)	1.0
Chronic respiratory diseases	0	4 (1.2)	1.0
Chronic kidney disease	1 (4.5)	1 (0.3)	0.118
Tumor	0	2 (0.6)	1.0
Autoimmune disease	0	6 (1.8)	1.0
Chronic hepatitis	0	2 (0.6)	1.0
Hematologic disease	0	2 (0.6)	1.0

All variables are expressed as *n* (%) except when indicated otherwise.

**Table 2:** Nasopharyngeal and oropharyngeal nucleic acid Ct values in the symptomatic and the asymptomatic groups.

	Symptomatic group	Asymptomatic group	*P*
Nasopharyngeal nucleic acid test Ct value	30.5 (24.5–35)	27.8 (23.4–34.5)	0.187
Oropharyngeal nucleic acid test Ct value	34.5 (30–37)	33.5 (26–35)	0.105

Values are expressed as median (interquartile range). Ct: cycle threshold.

**Table 3:** Nucleic acid test Ct values of the symptomatic and the asymptomatic groups stratified by oropharyngeal (OP) versus nasopharyngeal (NP) swabs and by vaccination status.

	Ct of symptomatic patients (NP)	Ct of symptomatic patients (OP)	Ct of asymptomatic patients (NP)	Ct of asymptomatic patients (OP)
Unvaccinated	32.25 (24.4–34.9)	35 (30–37)	34.5 (34.5–34.5)	35 (34.5–35)
Vaccinated	30.25 (25–35)	35 (30–37)	27.3 (22.1–34.4)	32 (25.8–35)
*P*	0.889	0.318	0.26	0.172

Values are expressed as median (interquartile range) Ct: cycle threshold.

## Data Availability

The data used to support the findings of this study are available from the corresponding author upon request.
